# Experiment of a Cut-Out Piezoelectric Beam Energy Harvester Under Wind-Induced Vibration

**DOI:** 10.3390/mi16040378

**Published:** 2025-03-27

**Authors:** Xuhong Fan, Chongming Zhao, Wenan Jiang

**Affiliations:** Faculty of Civil Engineering and Mechanics, Jiangsu University, Zhenjiang 212013, China; 1000002355@ujs.edu.cn (X.F.); zhaocm420@163.com (C.Z.)

**Keywords:** wind-induced vibration, cut-out piezoelectric beam, energy harvesting, bluff body

## Abstract

To address the low electromechanical conversion efficiency associated with traditional single-degree-of-freedom (SDOF) piezoelectric energy harvesters, this study proposes a two-degree-of-freedom (2-DOF) cut-out piezoelectric beam for wind-induced vibration energy harvesting. Experimental comparisons conducted on four bluff bodies indicated that the triangular column exhibits superior aerodynamic stability, achieving an output voltage of 11.6 V at a wind speed of 7.0 m/s. Furthermore, the cut-out piezoelectric beam demonstrated a 1.9-fold increase in output voltage compared to its non-cut-out counterpart. These results underscore the potential of the 2-DOF cut-out piezoelectric beam design as an autonomous power solution for IoT nodes operating in complex environments.

## 1. Introduction

Recent research conducted by the International Energy Agency (IEA) suggests that, by the year 2030, the global power demand of internet of things (IoT) terminal devices is expected to constitute 4.2% of total electricity consumption, with more than 30% of these devices deployed in remote or harsh environments [1]. As IoT devices experience exponential growth (i.e., surpassing 29 billion units worldwide by 2030) and increasingly integrate into critical infrastructures such as Industry 4.0 and smart cities, the challenge of ensuring a reliable energy supply has escalated to a global concern [2]. In recent years, the autonomous energy supply for distributed low-power electronic systems has emerged as a significant technical bottleneck that limits their large-scale applications [3,4]. However, previously underutilized environmental energy sources (e.g., solar, geothermal, wind, hydropower, and mechanical vibration energy) have increasingly become a focal point in global energy supply research [5,6,7].

Wind energy has emerged as one of the most promising sources of clean energy due to its environmental sustainability, renewable characteristics, widespread geographical availability, and substantial economic benefits [8,9]. Utilizing the principles of fluid–structure interaction (FSI) dynamics, wind-induced vibration energy harvesting technology is capable of converting stochastic wind energy into periodic mechanical vibrations within structures [10]. This mechanical energy is subsequently transformed into electricity through various electromechanical conversion mechanisms, including the piezoelectric effect (strain–charge coupling) [11], the magnetoelectric effect (stress–magnetic polarization coupling) [12], and the magnetostrictive effect (magnetic field–deformation coupling) [13]. Among these three mechanisms for converting wind-induced vibrations into energy, the piezoelectric conversion mechanism exhibits an energy density that is three times greater than both electromagnetic and electrostatic conversions, while also offering advantages such as miniaturization and cost-effectiveness [14].

The operational principle of piezoelectric wind energy harvesters is predicated on the coupled dynamics of FSI and multiphysics phenomena. Aerodynamic instabilities, including vortex-induced vibrations, galloping, and flutter mechanisms, facilitate the efficient transduction of airflow perturbations into electrical energy generation [15]. However, conventional designs are often constrained by limitations such as restricted operational bandwidth, suboptimal power density, and inadequate adaptability to variations in wind speed [16]. To address these challenges, targeted investigations have been conducted across various technical domains. Yang et al. [17] validated through experimental and numerical analyses that square cross-sectional geometries can achieve a threshold wind speed of 2.5 m/s and a peak power output of 8.4 mW. Yang et al. [18] explored the aerodynamic shape optimization of bluff bodies, demonstrating that symmetrical configurations with convex trailing edges and concave top and bottom surfaces can reduce critical wind speeds while enhancing power generation. Kang et al. [19] utilized a microscale cylindrical oscillator integrated with piezoelectric thin films fabricated through MEMS processes to establish a strong correlation between high-frequency vortex street dynamics and voltage signals. Song et al. [20] developed a width-tapered double-cantilever beam structure with asymmetric mass distribution to achieve broadband energy harvesting through bending–torsion coupled vibrations. He et al. [21] implemented a modal synergy mechanism that combines vortex-induced vibrations and buffeting effects, effectively merging energy capture intervals across operational wind speed ranges to create continuous high-efficiency output bands.

Recent studies have focused on the cross-sectional optimization of bluff bodies for SDOF configurations, as well as structural refinement for monolithic bluff body designs. However, SDOF piezoelectric energy harvesters that are integrated with bluff bodies face inherent limitations due to their cantilevered architectures, which impose intrinsic bandwidth constraints that are incompatible with the broadband characteristics of ambient wind fields. Emerging theoretical frameworks for 2-DOF systems offer promising alternatives. Wu et al. [22] developed a 2-DOF nonlinear energy harvester utilizing magnetic repulsion forces, effectively overcoming the narrowband limitations associated with SDOF counterparts in fluctuating wind conditions. Jiang et al. [23] incorporated an internal resonance mechanism within an electromagnetic harvesting system, validating synchronized oscillation phenomena through harmonic balance techniques. Quantitative assessments revealed enhancements of 92.81% in current amplitude and 153.86% in power density, relative to non-resonant baselines. Despite these advancements, empirical validation of 2-DOF systems has not kept pace with theoretical developments. Notably, broadband energy harvesting capabilities have been confirmed in mechanically vibrated cut-out piezoelectric beams [24]. This paper proposes a cut-out piezoelectric beam wind-induced vibration energy harvester by integrating a 2-DOF cut-out beam piezoelectric harvester with a galloping conversion mechanism. Through the synergistic configuration of a frame-type primary beam and a cantilever secondary beam, this design enhances the FSI effect while establishing a strain gradient amplification mechanism. Comparative experiments were conducted to evaluate the energy output performance between the cut-out piezoelectric beam and the SDOF non-cut-out piezoelectric beam. Experimental results indicate that within a wind speed range of 1.3~7.0 m/s, the proposed harvester achieves a root mean square (RMS) voltage amplitude of 11.6 V, representing enhancements of 90.1% compared to the non-cut-out counterpart. This breakthrough presents a technical pathway for the design of broadband wind energy harvesters.

## 2. Experimental Program

### 2.1. Experimental Model

**Figure 1:**
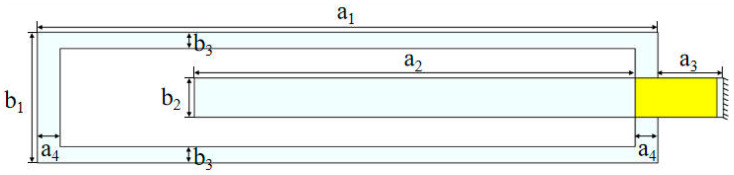
Cut-out piezoelectric beam energy harvester configuration.

This study presents a cut-out piezoelectric beam designed for harvesting wind-induced vibration energy, utilizing a 2-DOF cut-out piezoelectric beam configuration (Figure 1). The design achieves aerodynamic compatibility with the bluff body by extending the frame-type primary beam by 40 mm, while preserving the original length of the cantilever secondary beam within the frame. This configuration mitigates collision-induced nonlinear effects that may arise from potential relative motion between the secondary beam and the bluff body, thereby enhancing the stability of wind-induced vibration energy harvesting. Additionally, it incorporates a bimorph piezoelectric ceramic structure (PZT-5H) bonded at the junction of the beam, serving as the energy conversion unit. The mechanical energy generated by the excitation of the bluff body vibrations is transmitted to the piezoelectric ceramics through the coupled vibration modes of the primary and secondary beams, facilitating a high-efficiency conversion of vibrational energy into electrical energy.

**Figure 2:**
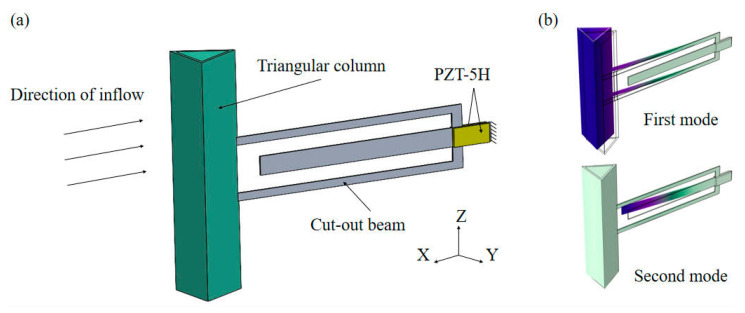
Schematic of cut-out piezoelectric beam wind-induced vibration energy harvester: (**a**) experimental model; (**b**) y-directional vibration modes.

**Figure 3:**
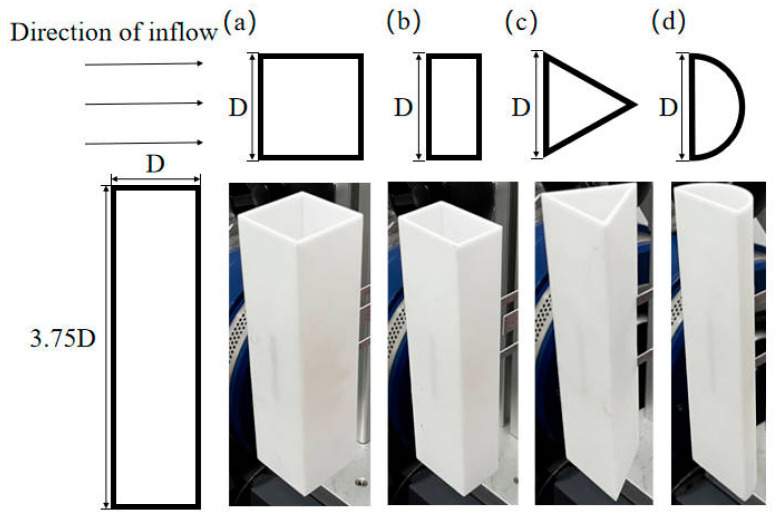
Four shapes of bluff bodies: (**a**) square column; (**b**) semi-square column; (**c**) triangular column; (**d**) semi-cylinder.

Figure 2a illustrates the experimental model of the cut-out piezoelectric beam harvester, which consists of a bluff body, a cut-out beam, and a bimorph PZT-5H. A modal analysis of the cut-out beam was performed using COMSOL 6.2 Multiphysics, with the results depicted in Figure 2b. The y-directional dual-mode characteristics of the beam facilitate a 2-DOF energy harvesting mechanism. The bluff body, which functions as the primary aerodynamic component of the harvester, significantly impacts its galloping characteristics through its geometric configuration. This study specifically selects four representative bluff body shapes for experimental evaluation (Figure 3) to examine the effect of cross-sectional shapes on energy harvesting efficiency. All bluff bodies are designed to have identical windward areas to ensure comparability in the experiments, with the baseline dimensions defined as the windward width (D=40 mm) and length (L=150 mm) of the bluff body. Under consistent Reynolds number conditions, the various bluff bodies alter flow field characteristics, including boundary layer separation points and vortex shedding frequencies, as a result of their different cross-sectional morphologies [25,26,27]. The wind-induced vibration experiments conducted on the different bluff bodies in this study were performed within the subcritical Reynolds number regime ($Re=\frac{UD}{v}=3.5\times 10^{3}\sim 18.7\times 10^{3}$), achieved by regulating the wind speed between 1.3 m/s and 7.0 m/s while maintaining constant characteristic length (D=40 mm) and air kinematic viscosity ($v\approx 1.5\times 10^{-5}m^{2}/s$).

### 2.2. Experimental Configurations

**Figure 4:**
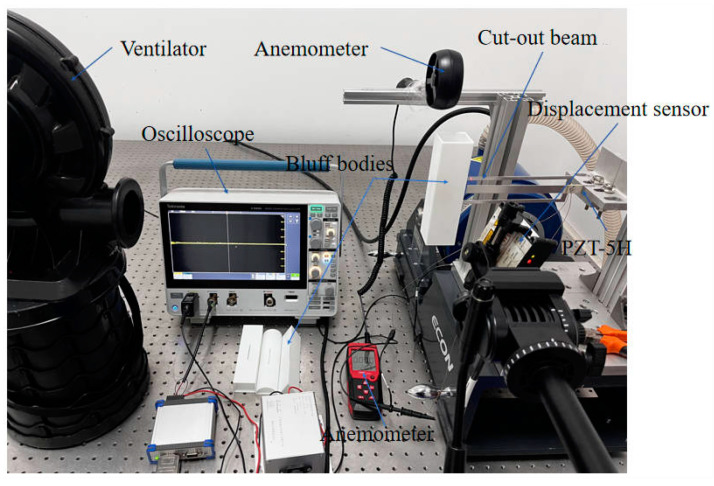
Cut-out piezoelectric beam wind-induced vibration energy harvester experimental configuration.

Figure 4 presents the experimental configurations of the cut-out piezoelectric beam designed for wind-induced vibration energy harvesting, with the geometric parameters in Table 1. The system utilizes an axial flow fan as the primary power source, which produces a continuously adjustable and stable airflow ranging from 1.3 m/s to 7.0 m/s by varying the distance to the bluff body (2~8 m). The real-time average wind speed is monitored using an anemometer. The harvester is affixed at its fixed end to a rigid base, while the free end is integrated into the bluff body connection and symmetrically bonded with PZT-5H bimorph piezoelectric ceramics near the fixed end. When uniform incoming flow in the x-direction interacts with the bluff body, its aerodynamic geometry induces flow separation, resulting in self-excited y-directional galloping motion. The geometric lever amplification mechanism of the cut-out beam transforms micro-amplitude displacements into localized high strain, facilitating mechanical-to-electrical energy conversion through the direct piezoelectric effect. Finally, a displacement sensor accurately records the vibration displacements at the tip of the cantilever secondary beam, while the piezoelectric outputs are transmitted using copper wires to an oscilloscope for voltage signal acquisition.

## 3. Comparison and Analysis of Experimental Results

### 3.1. The Optimal Bluff Body

**Figure 5:**
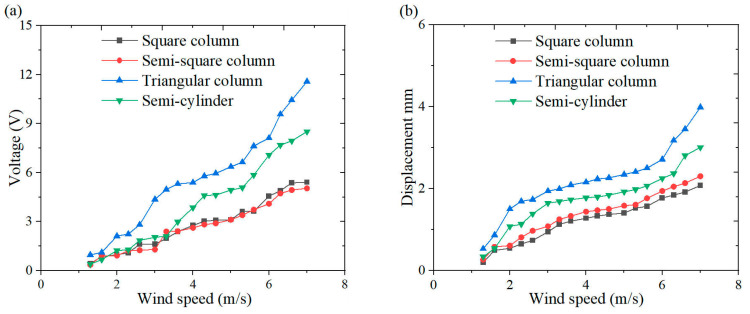
Output response of cut-out piezoelectric beam wind-induced vibration energy harvester under different bluff bodies: (**a**) voltage wind speed curve; (**b**) displacement wind speed curve.

An anemometer was installed upstream of the collector to monitor freestream velocity in real time, thereby ensuring flow uniformity throughout the experimental campaign. The inherent stochasticity associated with vortex generation, merging, and dissipation processes within the wake region induces non-stationary alternating voltage signals in PZT-5H. To address the effects of phase randomness while characterizing effective electrical power output, RMS voltage is employed as the primary evaluation metric, providing a time-averaged quantification of voltage conversion levels [28]. Experiments were conducted by controlling wind speeds within the range of 1.3~7.0 m/s, revealing nonlinear growth trends of RMS voltage and displacement in relation to flow velocity for the four bluff bodies (Figure 5). Notably, the triangular column bluff body exhibits superior broadband energy harvesting performance, achieving 11.6 V and 3.9 mm at a wind speed of 7.0 m/s.

**Figure 6:**
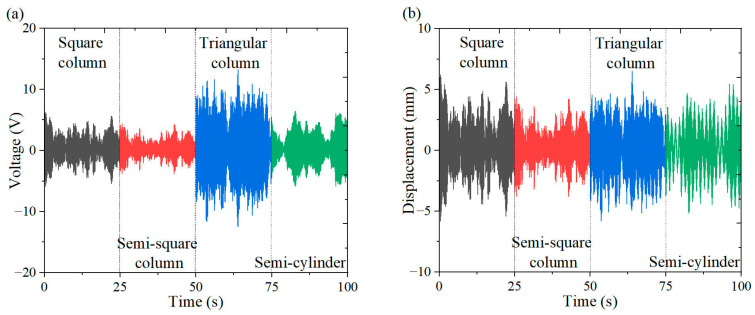
Output response of different bluff bodies at 3 m/s: (**a**) voltage time curve; (**b**) displacement time curve.

**Figure 7:**
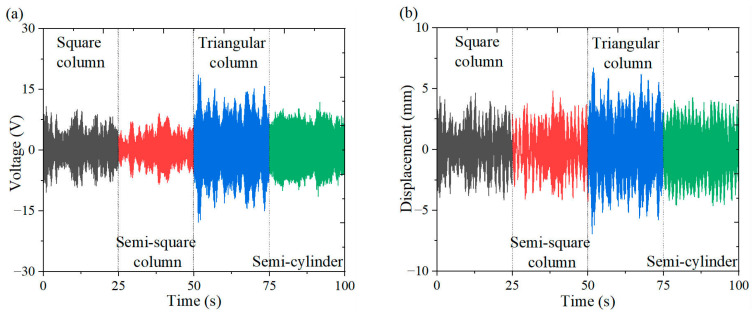
Output response of different bluff bodies at 5 m/s: (**a**) voltage time curve; (**b**) displacement time curve.

**Figure 8:**
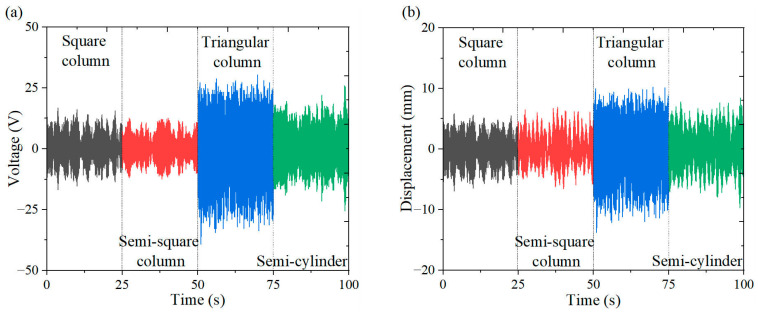
Output response of different bluff bodies at 7 m/s: (**a**) voltage time curve; (**b**) displacement time curve.

Figure 6, Figure 7 and Figure 8 illustrate the energy output and tip displacements of the cantilever secondary beam associated with the cut-out piezoelectric beam designed for wind-induced vibration energy harvesting, evaluated under four bluff body configurations: square column, semi-square column, triangular column, and semi-cylinder. The time-domain plots, recorded at varying wind speeds, indicate that the triangular column exhibits a lower amplitude of output voltage fluctuations and a more concentrated signal distribution. Notably, the triangular cross-section, characterized by its sharp geometric edges, demonstrates superior flow separation properties in comparison to the other bluff bodies. Flow separation occurs along the trailing single-edge corner of the triangular cross-section, resulting in an asymmetric yet stabilized flow path with fewer separation points than those observed in the other bluff body configurations. This mode of single-edge separation enhances the periodicity of vortex shedding and promotes resonant coupling between the dynamics of the vortices and the structural vibration frequencies.

### 3.2. Power Output and Optimal Resistance

**Figure 9:**
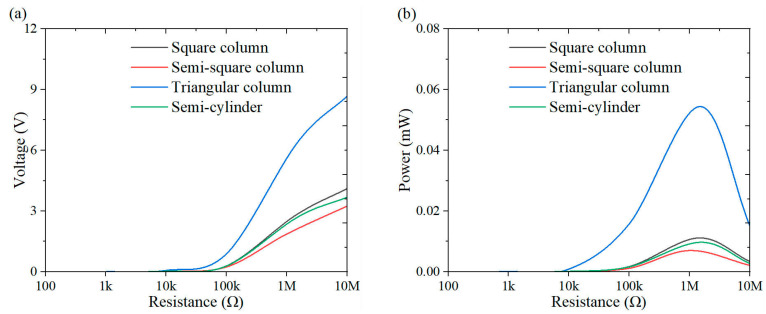
Response curves for different resistive loads at 3 m/s: (**a**) voltage resistance curve; (**b**) power resistance curve.

**Figure 10:**
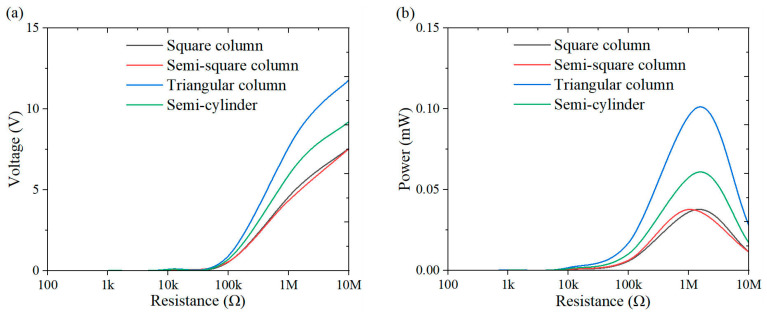
Response curves for different resistive loads at 5 m/s: (**a**) voltage resistance curve; (**b**) power resistance curve.

**Figure 11:**
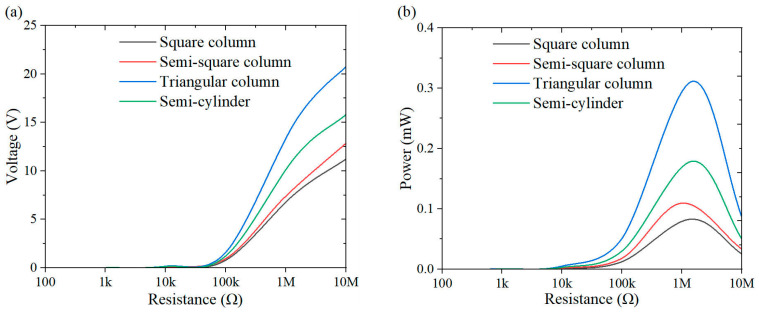
Response curves for different resistive loads at 7 m/s: (**a**) voltage resistance curve; (**b**) power resistance curve.

**Figure 12:**
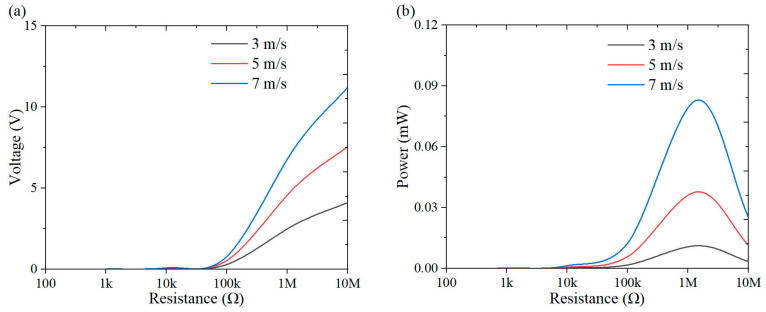
Response curves of square column under variable resistive loads and wind speeds: (**a**) voltage resistance curve; (**b**) power resistance curve.

**Figure 13:**
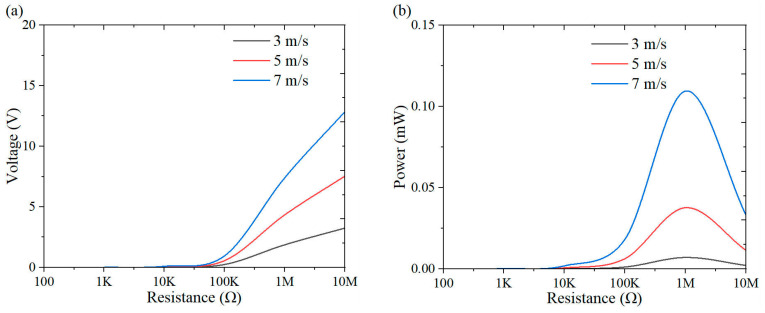
Response curves of semi-square column under variable resistive loads and wind speeds: (**a**) voltage resistance curve; (**b**) power resistance curve.

**Figure 14:**
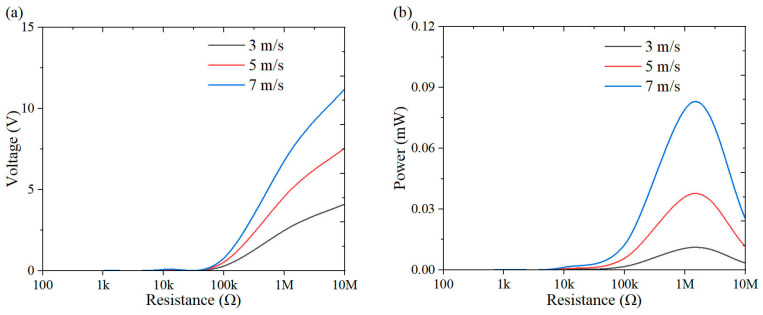
Response curves of triangular column under variable resistive loads and wind speeds: (**a**) voltage resistance curve; (**b**) power resistance curve.

**Figure 15:**
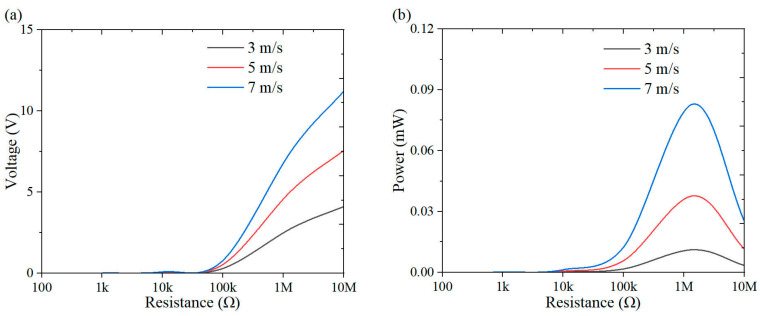
Response curves of semi-cylinder under variable resistive loads and wind speeds: (**a**) voltage resistance curve; (**b**) power resistance curve.

This section investigates the effect of load resistance on the output response of the system, with a specific focus on power performance. Figure 9, Figure 10, Figure 11, Figure 12, Figure 13, Figure 14 and Figure 15 depict the variations in output voltage and power in relation to load resistance for various bluff bodies subjected to wind speeds of 3.0 m/s, 5.0 m/s, and 7.0 m/s. As load resistance increases, the output voltage demonstrates a monotonic increase, whereas the power output exhibits a characteristic rise-and-fall trend. The optimal load resistance values vary among the different bluff bodies. The square column achieves peak power outputs of 3.4 μW, 11.4 μW, and 25 μW at a load resistance of 1.4 MΩ. The semi-square column reaches maximum power outputs of 6.7 μW, 37.7 μW, and 109.5 μW at 1 MΩ. The triangular column attains maximum power outputs of 54.4 μW, 101.1 μW, and 311.4 μW at 1.5 MΩ. Similarly, the semi-cylinder configuration achieves peak power outputs of 9.7 μW, 60.9 μW, and 178.9 μW at its optimal resistance of 1.5 MΩ.

## 4. Comparative Analysis of Energy Harvesting Performance in Cut-Out and Non-Cut-Out Piezoelectric Beams Under a Triangular Column

### 4.1. Non-Cut-Out Piezoelectric Beams Analysis

**Figure 16:**
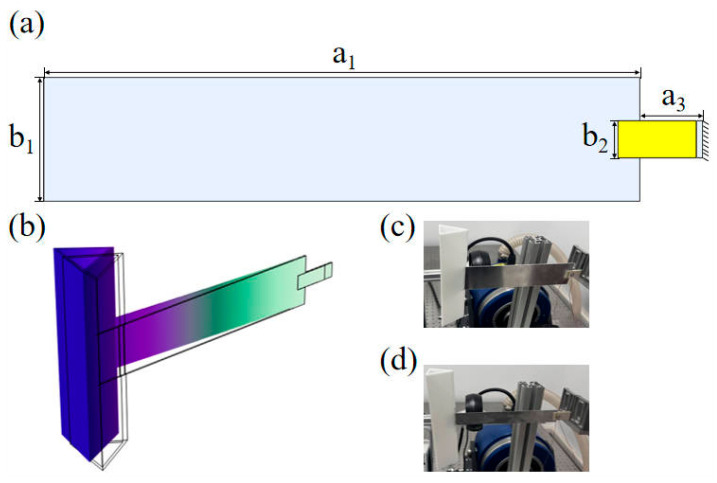
Non-cut-out piezoelectric beam harvester model: (**a**) parameter; (**b**) vibration mode; (**c**) b_1_ = 40 mm; (**d**) b_1_ = 20 mm.

This section presents experimental research on the energy harvesting performance of cut-out versus non-cut-out piezoelectric beams under a triangular column, with a focus on a comparative analysis of energy conversion efficiency. Figure 16 illustrates the parameters, vibration mode, and experimental models of the non-cut-out piezoelectric beams, wherein the PZT-5H material is affixed at the same location as in the cut-out beam design. This consistent baseline reference for energy conversion units mitigates interference arising from layout variations of the piezoelectric elements during performance comparisons.

**Figure 17:**
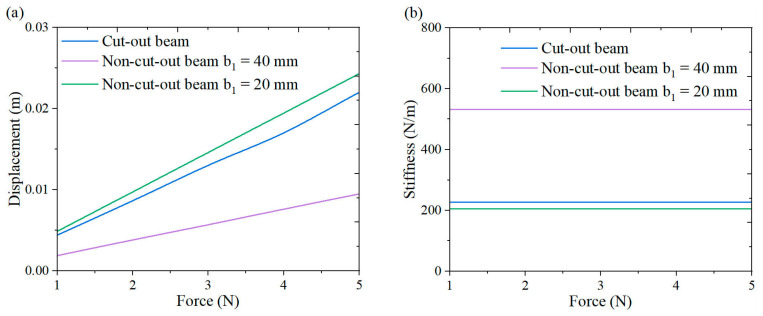
Stiffness Comparison: (**a**) displacement force curve; (**b**) stiffness force curve.

To evaluate the output performance of the cut-out beam, comparative experiments were conducted utilizing two non-cut-out beam configurations: one that matched the width of the cut-out beam and another that featured a reduced width with geometrically tailored stiffness equivalent to the cut-out beam. Figure 17 depicts the stiffness comparison of these three beam configurations.

### 4.2. Experimental Results

**Figure 18:**
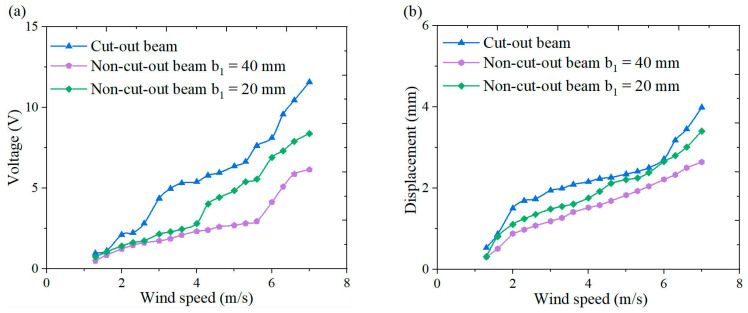
Comparison of the outputs from cut-out piezoelectric beam and non-cut-out piezoelectric beam wind-induced vibration energy harvesters under a triangular column: (**a**) voltage wind speed curve; (**b**) displacement wind speed curve.

**Figure 19:**
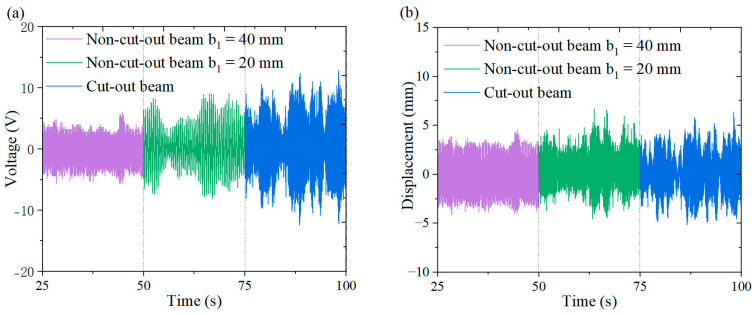
Comparison of the outputs from wind-induced vibration harvesters for cut-out piezoelectric beam and non-cut-out piezoelectric beam at 3 m/s under a triangular column: (**a**) voltage time curve; (**b**) displacement time curve.

**Figure 20:**
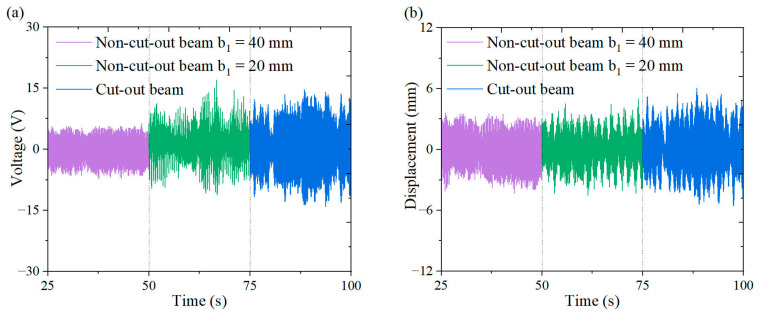
Comparison of the outputs from wind-induced vibration harvesters for cut-out piezoelectric beam and non-cut-out piezoelectric beam at 5 m/s under a triangular column: (**a**) voltage time curve; (**b**) displacement time curve.

**Figure 21:**
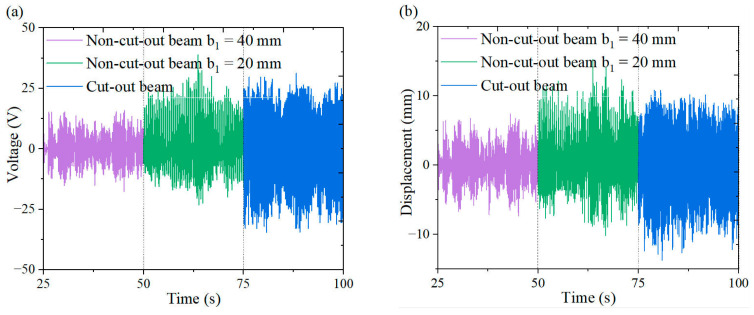
Comparison of the outputs from wind-induced vibration harvesters for cut-out piezoelectric beam and non-cut-out piezoelectric beam at 7 m/s under a triangular column: (**a**) voltage time curve; (**b**) displacement time curve.

Figure 18, Figure 19, Figure 20 and Figure 21 illustrate the energy harvesting performance of cut-out versus non-cut-out piezoelectric beams across a wind speed range of 1.3~7.0 m/s, highlighting the superior performance of the cut-out configuration. Experimental data indicate that, at a wind speed of 7.0 m/s, the cut-out piezoelectric beam generates an output voltage of 11.6 V, which represents enhancements of 90.2% and 38.1% when compared to non-cut-out beams with widths of 40 mm (6.1 V) and 20 mm (8.4 V), respectively. Although the width-reduced beam achieves comparable stiffness, it produces a voltage that is 38.1% lower. The reduced stiffness facilitates resonance at lower wind speeds. However, it necessitates geometric optimization to prevent excessive compliance. These comparative results substantiate the amplified strain gradient effects resulting from the different stress concentration topology of the cut-out beam, as well as its improved aerodynamic energy capture efficiency.

## 5. Discussion

As a critical component of environmental energy harvesting systems, wind-induced vibration energy conversion systems demonstrate multidimensional technological advantages. Their non-intrusive ecological characteristics ensure compatibility with fragile ecosystems, while their inherent dynamic renewability addresses the temporal limitations associated with conventional power supply paradigms. Additionally, their broad applicability removes geographical constraints on system deployment. Engineering practices have shown that, through structural innovation in wind-induced vibration mechanisms and topological optimization of energy conversion mechanisms, miniaturized wind-induced vibration energy harvesters can sustain the continuous operation of electronic devices, thereby providing a theoretical foundation for the construction of autonomous energy networks. This study responds to the demand for broadband, high-efficiency energy conversion in wind-induced vibration energy harvesters by designing a 2-DOF cut-out piezoelectric beam harvester based on the aerodynamic–mechanical synergistic effect, resulting in a significant enhancement in energy conversion efficiency. The key conclusions are outlined as follows:

(1) Within the wind speed range of 1.3~7.0 m/s, the triangular column demonstrates enhanced energy harvesting performance in comparison to the square column, semi-cylinder, and semi-square column. At a wind speed of 7.0 m/s, the output voltage of the triangular column attains a value of 11.6 V.

(2) A comparative experiment involving non-cut-out piezoelectric beams demonstrates that the cut-out piezoelectric beam achieves enhancements in voltage outputs of 190.1% and 138%, respectively, at a velocity of 7.0 m/s. This result validates the superior aerodynamic energy capture efficiency of the cut-out piezoelectric beam design.

Based on the developments in aerodynamic–mechanical synergistic optimization and the innovative design of 2-DOF cut-out beam structures, future research should focus on the practical development of energy harvesting from wind-induced vibrations through the following:

(1) Establishing a fluid–structure–electric multiphysics coupled simulation model that integrates dynamic mesh techniques, piezoelectric constitutive equation coupling methods, and microstructured surface designs on triangular column bluff bodies to enhance synergistic energy capture from vortex-induced vibration and galloping;

(2) Developing bistable structures with wind speed-adaptive capabilities via magnetic-controlled variable stiffness mechanisms to address the broadband time-varying characteristics of natural wind fields.

## Figures and Tables

**Table 1:** Material parameters.

Parameters	Value
Cut-out beam	
Young’s modulus/GPa	70
Poisson’s ratio	0.33
Density/(kg·m^−3^)	2700
Length a_1_/mm	190
Length a_2_/mm	135
Length a_3_/mm	20
Length a_4_/mm	7
Width b_1_/mm	40
Width b_2_/mm	12
Width b_3_/mm	5
Thickness/mm	1
PZT-5H	
Density/(kg·m^−3^)	7500
Length/mm	25
Width/mm	12
Thickness/mm	0.2
Piezoelectric constant	1704.4/1704.4/1 433.6

## Data Availability

The original contributions presented in this study are included in the article. Further inquiries can be directed to the corresponding author.
